# Anti-CD47 Antibody Enhances the Efficacy of Chemotherapy in Patients with Gastric Cancer Liver Metastasis

**DOI:** 10.7150/jca.80725

**Published:** 2023-01-16

**Authors:** Zhaorui Liu, Huiying Chen, Na Ta, Zheng Shi, Lu Zhan, Ting Han, Jinghui Zhang, Xusheng Chang, Kai Yin, Mingming Nie

**Affiliations:** 1 Department of Gastrointestinal Surgery, The First Affiliated Hospital of Naval Medical University, Shanghai 200433, China.; 2 Department of Pathogen Biology, College of Basic Medical Sciences of Naval Medical University, Shanghai 200433, China.; 3 Department of Pathology, The First Affiliated Hospital of Naval Medical University, Shanghai 200433, China.

**Keywords:** CD47 expression, gastric cancer liver metastasis, phagocytic activity of Kupffer cells

## Abstract

Patients with gastric cancer liver metastasis (GCLM) are often treated with palliative care, and they show a poor prognosis. In gastric cancer, high CD47 expression has been shown to indicate a poor prognosis. CD47, expressed on the cell surface, prevents the cells from being phagocytosed by macrophages. Anti-CD47 antibodies have been shown to be effective in the treatment of metastatic leiomyosarcoma. Nonetheless, the role of CD47 in GCLM has not yet been elucidated. Here, we showed that CD47 expression in GCLM tissues was higher than that *in situ*. Moreover, we demonstrated that high CD47 expression correlated with an adverse prognosis. Accordingly, we investigated the role of CD47 in the development of GCLM in mouse liver. Knockdown of* CD47* inhibited GCLM development. Furthermore, *in vitro* engulfment assays showed that decreased CD47 expression led to an increased phagocytic activity of Kupffer cells (KCs). Using enzyme-linked immunosorbent assay, we determined that *CD47* knockdown promoted cytokine secretion by macrophages. Furthermore, we found that tumor-derived exosomes decreased KC-mediated phagocytosis of gastric cancer cells. Finally, in a heterotopic xenograft model, the administration of anti-CD47 antibodies inhibited tumor growth. In addition, as 5-fluorouracil (5-Fu)-based chemotherapy is the cornerstone in GCLM treatment, we administered a combination of anti-CD47 antibodies and 5-Fu, which acted synergistically to suppress the tumor. Overall, we demonstrated that tumor-derived exosomes are involved in GCLM progression, targeting CD47 inhibits gastric cancer tumorigenesis, and a combination of anti-CD47 antibodies and 5-Fu shows potential for treating GCLM.

## Introduction

Gastric cancer (GC) is the fifth most common type of cancer worldwide, with 108.9 million new cases and 76.9 million deaths in 2020 1. Approximately 50% of the world's GC cases and deaths are in China, with metastasis being the leading cause of death 2. The most common metastatic sites are the liver (48%), peritoneum (32%), lungs (15%), and bones (12%) 3. According to the current guidelines, the standard treatment for GC liver metastasis (GCLM) is systemic chemotherapy 4. In the past few decades, 5-fluorouracil (5-Fu)-based chemotherapy has been the main adjuvant therapy for GC. Some patients initially respond to 5-Fu-based chemotherapy, but later develop drug-resistant relapses, leading to cancer progression and death 5. Despite the development of novel biomarkers, the prognosis of GC remains unsatisfactory. Therefore, studies aimed at identifying and developing new therapeutics against GCLM are urgently required to improve its prognosis.

The high frequency of liver metastasis can be partially explained by the rich circulation, anatomical features, and immune system of the liver 3. Kupffer cells (KCs) account for approximately 80% of the tissue-resident macrophages in the liver 6. These cells play an important role in phagocytosis as well as the detection and elimination of tumor cells and other antigens that pass through the liver sinusoids 7. To evade phagocytosis by macrophages, many cancer cells express high levels of CD47 8,9. CD47 functions as a “don't eat me” signal and is widely expressed on the surface of various cell types; this protein can bind to macrophage SIRPα to inhibit phagocytosis 10. Previous studies have shown that CD47 expression is higher in GC than in other, adjacent cancers, and that high CD47 expression indicates a poor prognosis 11,12. Furthermore, *in vitro* phagocytosis of cancer cells by human macrophages has been shown to be enhanced when monoclonal antibodies were used to block CD47 8,13. Moreover, preclinical studies have revealed that blocking CD47 inhibits solid tumor growth and enhances the efficacy of conventional chemotherapy, radiation therapy, and some targeted cancer therapies 13-15. Tumor-derived exosomes have been shown to be involved in the phagocytosis by macrophages. Zhang et al. validated the pre-metastatic role of GC-derived exosomes in the formation of hospitable liver microenvironment. Moreover, tumor-derived exosomes have been shown to determine the propensity for metastasis and organ sites of future metastasis 16,17. Despite these findings, the role of CD47 in GCLM has not yet been reported.

We hypothesized that high CD47 expression would suppress the growth of GC in the liver by inhibiting phagocytosis of cancer cells by KCs. Additionally, we speculated that targeting CD47 can inhibit GCLM, and its combination with chemotherapy drugs may enhance this effect. In this study, we first evaluated the expression of CD47 in GCLM. We then investigated the effect of *CD47* knockdown on tumorigenesis in mice *in vivo* and on the phagocytosis of cancer cells by KCs *in vitro*. In addition, we explored the role of tumor-derived exosomes in GCLM. Finally, we evaluated the potential of targeting CD47 in GCLM treatment. Preclinical evidence gathered in our study shows that CD47 is an effective target in the treatment of GCLM, and its combination with 5-Fu can improve the efficacy of 5-Fu-based chemotherapy against GCLM.

## Materials and Methods

### GCLM tissues

Ten GC cases with liver metastasis were recorded between 2019 and 2020 at the First Affiliated Hospital of Second Military Medical University (Shanghai, PRC). The surgical specimens of GCLM from the patients were prepared in a freezing environment. Written informed consent was obtained from all patients.

### Immunohistochemistry

To evaluate the *in situ* localization and expression patterns of CD47 in GCLM, we performed immunohistochemical staining of tissues obtained from patients with GCLM. Formalin-fixed paraffin-embedded blocks were subjected to serial sectioning, and the sections (3-μm thick) were stained with hematoxylin and eosin. The sections were incubated with anti-CD47 antibodies (0.2 μg/mL; 1:100; Cat# ab3283, RRID:AB_303671; Abcam, Cambridge, UK) overnight at 4 °C followed by incubation with a peroxidase-labeled polymer for 120 min at 25 °C.

### RNA preparation and quantitative real-time PCR

Total RNA from human primary GC spheres or metastatic liver was extracted with TRIzol™ (Life Technologies, Carlsbad, CA, USA) according to the manufacturer's instructions. *CD47* mRNA expression in clinical samples was detected in triplicate using real-time PCR with specific primers. The mRNA level of glyceraldehyde-3-phosphate-dehydrogenase (*GPDH*) was used as the internal standard. cDNA was generated from 1 μg of total RNA using SuperScript™ II Reverse Transcriptase (Life Technologies) and random hexamers. Quantitative real-time PCR was performed using the SYBR Green PCR Master Mix (Life Technologies) according to the manufacturer's instructions. The primer sequences were as follows: 5′-GAAGGTGAAACGATCATCGAGC-3′ and 5′-AATACCAAACTGTCCCAGAAC-3′ for *CD47* and 5′-GCACCACCAACTGCTTA-3′ and 5′-AGTAGAGGCAGGGATGAT-3′ for *GPDH*.

### Public dataset analysis

The public datasets GSE26899 and GSE26901 were obtained from the Gene Expression Omnibus (https://www.ncbi.nlm.nih.gov/geo/). The median of CD47 expression was determined as the cut-off value.

### Isolation of KCs

*BALB/c* nude mouse livers were mechanically dissociated using the gentleMACS™ Dissociator (#130-1-5-807; Miltenyi Biotec, Gladbach Bergisch, Germany). Next, the extracellular matrix was enzymatically degraded to prepare single-cell suspensions; the tissues remained structurally intact. The mouse livers were enzymatically digested using kit components, and the anti-F4/80 MicroBeads kit (Cat# 130-110-443, RRID: AB_2858241; Miltenyi Biotec) was used to isolate KCs according to the manufacturer's protocol.

### *In vitro* phagocytosis assay

To perform *in vitro* phagocytosis assays, we added 5 × 10^4^ liver-derived KCs to 24-well plates and co-cultured them with short hairpin (sh)-CD47 MKN45 or sh-NC MKN45 cells. The phagocytic index was calculated as the number of cells identified by phagocytosis per 100 KCs. For phagocytosis, KCs were co-cultured with sh-CD47 MKN45 cells, sh-NC cells, sh-CD47 MKN45 cells + exosomes (sh-NC), and sh-NC MKN45 cells + exosomes (sh-CD47).

### Cell lines and their culture

The human GC cell line MKN45 (CCTCC Cat# GDC0220, RRID: CVCL_0434) was purchased from the Type Culture Collection of the Chinese Academy of Science (Shanghai, PRC) and cultured in Dulbecco's modified Eagle's medium (Sigma-Aldrich, St. Louis, MO, USA) supplemented with 10% fetal bovine serum at 37 °C under 5% CO_2_ and constant humidity. MKN45 cells were transfected with lentivirus packaged with pLVX-Luc, PMD2G, and psPAX2 plasmids and cultured for 48 h. Subsequently, a stable luciferase-expressing cell line was selected using G418.

### Isolation of small extracellular vesicles

The supernatants of *CD47*- and *NC*-knockout MKN45 cells were centrifuged at 2000 × *g* for 30 min at 4 °C. For the removal of large vesicles, the supernatant was centrifuged at 10 000 × *g* for 45 min at 4 °C. To collect the filtrate, we passed the supernatant through a 0.45 µm filter membrane and then centrifuged the filtrate at 100 000 × *g* for 70 min at 4 °C. The resulting supernatant was removed, re-suspended in 10 mL of pre-cooled 1× phosphate-buffered saline (PBS), and centrifuged at 100 000 × *g* for 70 min at 4 °C. The supernatant was removed and resuspended in 100 μL of pre-cooled 1× PBS, and the exosomes were frozen at -80 °C. The size distribution of exosomes was determined using the Zetasizer HS III system (Malvern Instruments, Malvern, UK).

### Flow cytometry and cytokine detection

The BD FACSAria™ III flow cytometer (BD Biosciences, Franklin Lakes, NJ, USA) was used to measure the expression of the exosome biomarkers CD63 and CD9. Antibodies were incubated with the exosomes for 30 min at 37 °C. Next, the samples were centrifuged at 110 000 × *g* for 70 min at 4 °C, the supernatant was carefully removed, and 1 mL of pre-cooled PBS was added to the sample. The solution was centrifuged at 110 000 × *g* for 70 min at 4 °C, followed by flow cytometric analysis on a BD FACSAria™ cell sorter (BD Biosciences). Cytokines tumor necrosis factor α (TNF-α), interleukin 6 (IL-6) and IL-1β were detected using enzyme-linked immunosorbent assay (ELISA) kits (PT512, PI326 and PI301, respectively; Beyotime, Nanjing, PRC).

### Xenograft models

Animal maintenance and experiments were approved by the Institutional Animal Care and Use Committee of the First Affiliated Hospital of Second Military Medical University. Female *BALB/c* nude mice were purchased from the Center for Experimental Animals of the Chinese Academy of Sciences (Shanghai, PRC) and provided food and water *ad libitum*. To establish the heterotopic xenograft model, we suspended sh-CD47 MKN45-luc2 cells in PBS mixed with Matrigel, and 1 × 10^7^ cells were implanted into the liver of 6-week-old *BALB/c* nude mice. After 3 weeks, luciferin bioluminescence was measured. For the liver metastasis xenograft model of GC, MKN45-luc2 cells were injected into the spleen of 6-week-old *BALB/c* nude mice. After 1 week, the mice were injected three times per week with 400 µg of anti-CD47 antibodies (B6H12, Cat# 14-0479-82, RRID: AB_837143; Thermo Fisher Scientific, Waltham, MA, USA) and 5-Fu three times per week or a combination of B6H12+5-Fu or IgG (control) for 2 weeks through the tail vein. Bioluminescence images were acquired after the third week to confirm the presence of tumors.

### Bioluminescence imaging

An IVIS system (Caliper Life Science, Hopkinton, MA, USA) with Living Image software (version 4.0) was used to visualize* in vivo* bioluminescence. The maximum radiance was measured and the total flux (photos/s) was calculated for the delineated area of interest.

### Statistical analyses

The results for continuous variables are presented as mean ± standard deviation, and significance was determined using Mann‒Whitney test. A one-way analysis of variance (ANOVA) was used to compare the groups. The engulfment percentage under different treatments was compared using paired *t*-tests. A one- or two-way ANOVA was used to compare more than two groups. Survival analysis was performed using the Kaplan‒Meier, log rank test. In all statistical analyses, the threshold *P*-value was set at 0.05. GraphPad Prism version 8.00 software (GraphPad, Inc., La Jolla, CA, USA) was used for all testing and statistical analyses.

## Results

### CD47 shows higher expression in GC metastatic liver than *in situ*

**Figure 1:**
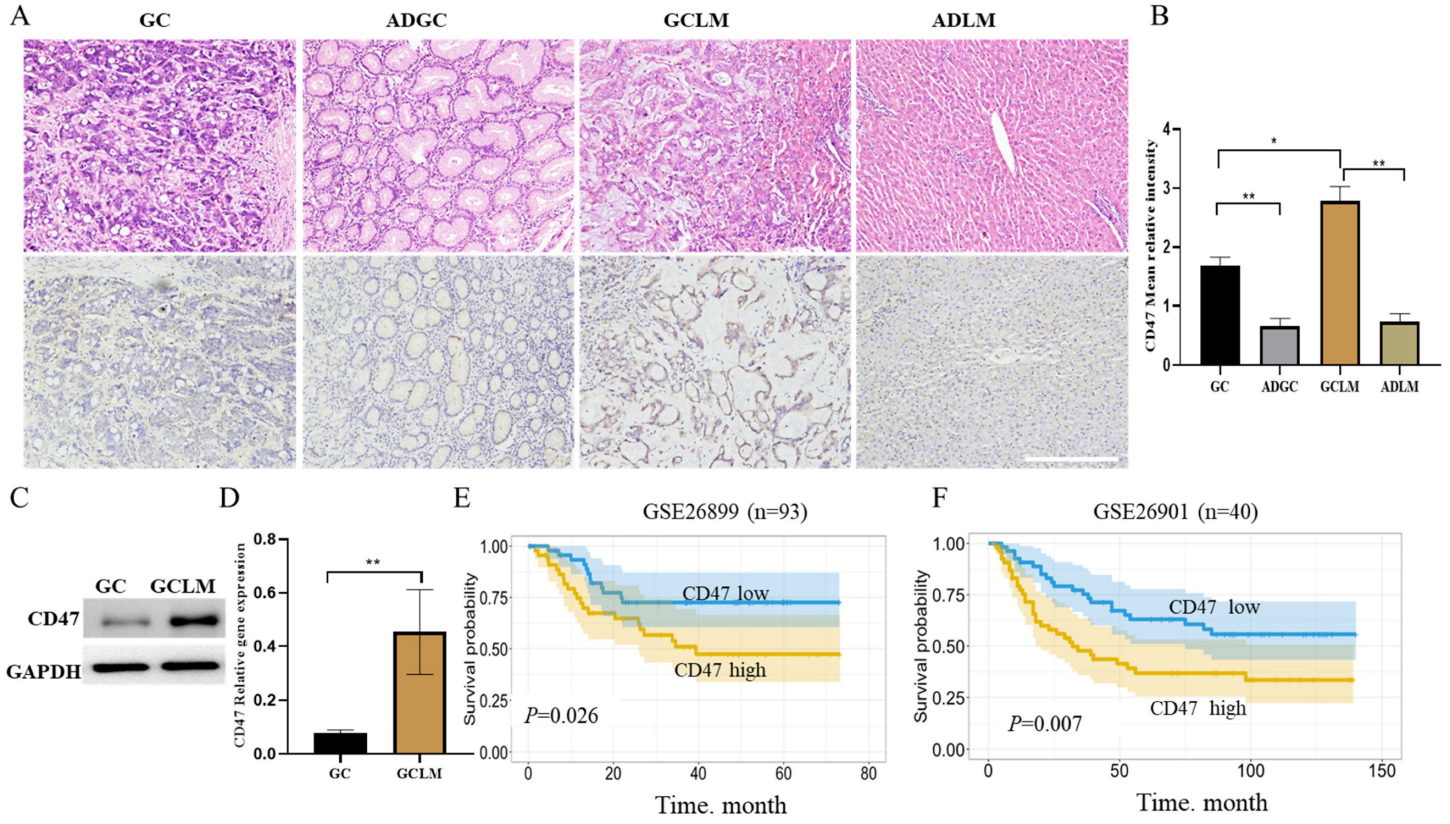
** CD47 expression is higher in metastatic liver than *in situ*.** (**A**) Images of hematoxylin and eosin- and CD47-stained GC, metastatic liver, and the corresponding para-carcinoma tissues. (**B**) Mean CD47 relative intensity values were determined from 10 samples of GC, metastatic liver, and the corresponding para-carcinoma tissues (n = 10, *P < 0.01, **P < 0.001, one-way ANOVA). (**C**) CD47 protein expression on the surface of GC metastatic liver evaluated using western blotting. The level of CD47 was higher in metastatic liver than in GC (n = 10, *P < 0.01). (**D**) RT-qPCR analysis of CD47 in GC and matched metastatic liver. *CD47* mRNA expression was higher in metastatic liver than in GC (n = 10, **P < 0.001, paired *t-*test). (**E, F**). Kaplan-Meier curves of overall survival values according to CD47 expression in GSE26899 (n = 93, log rank test) and GSE26901 (n = 40, log rank test) datasets. Abbreviations: **GC**: gastric cancer, **ADGC**: adjacent normal gastric tissue, **GCLM**: gastric cancer liver metastasis, **ADLM**: adjacent normal liver tissue.

Firstly, we evaluated CD47 expression in GC, metastatic liver, and the corresponding para-carcinoma using immunohistochemistry. The highest CD47 expression was observed in the metastatic liver, followed by GC tissues, in which CD47 expression was considerably higher than that in the corresponding para-carcinoma tissue (Figure 1A, B). Next, we compared CD47 expression between GCLM and GC tissues using western blotting and quantitative real-time PCR. The results showed that CD47 mRNA and protein expression was significantly higher in the metastatic tissues than in the primary GC tissues (**P < 0.01; Figure 1C, D). To confirm the prognostic value of CD47 expression in GC, we assessed the datasets GSE26899 and GSE26901. Kaplan-Meier curve and log rank test were used to compare the overall survival (OS) of patients with low and high CD47 expression. The results showed that patients with high CD47 expression exhibited shorter OS (*P* < 0.05, Figure 1E, F). Our assessment of the data revealed that CD47 was highly expressed in metastatic liver than in the normal tissue counterparts, thereby serving as an indicator of an adverse prognosis in GCLM.

### *CD47* knockdown inhibits GC growth in mouse liver tissues

**Figure 2:**
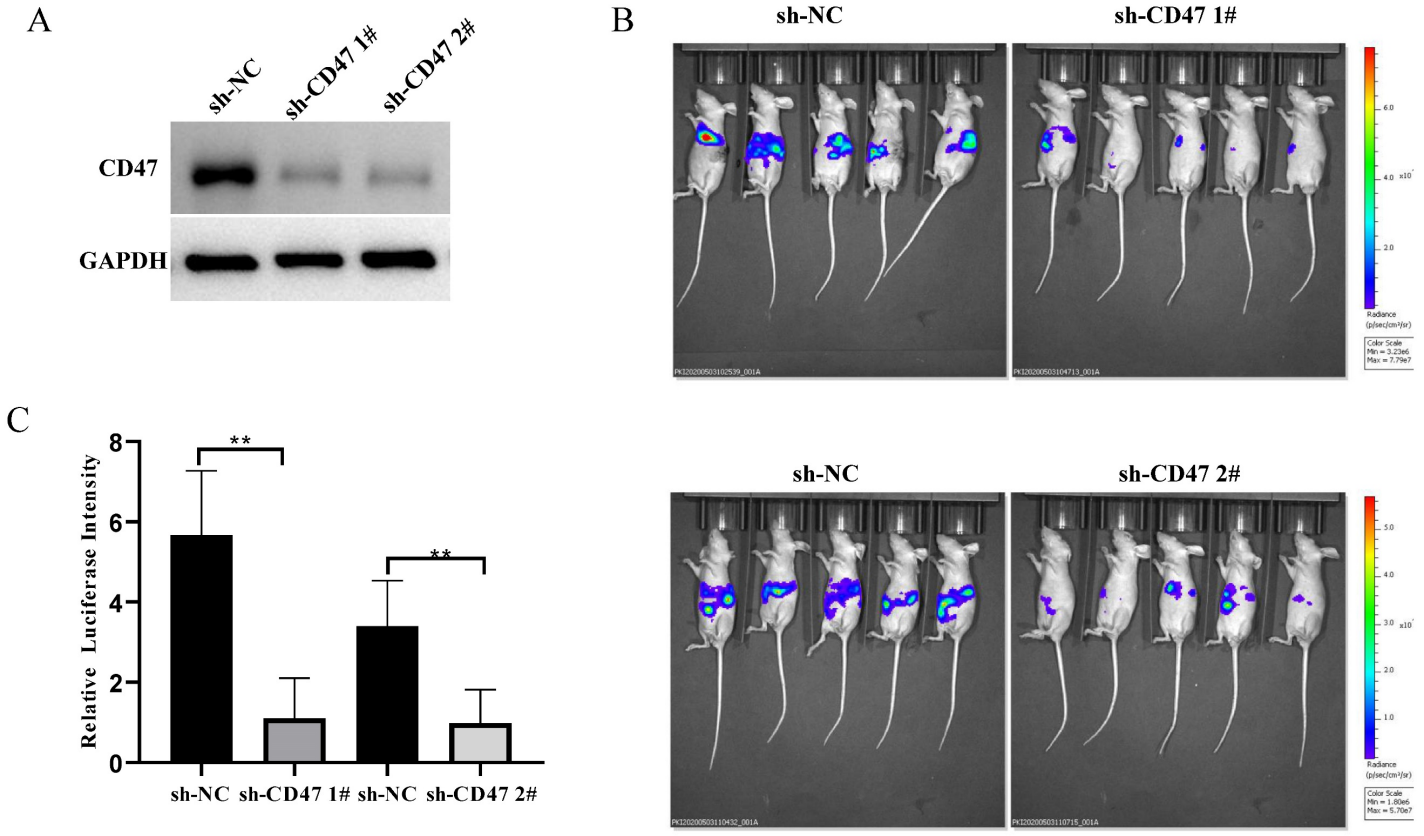
** Knockdown of *CD47* inhibits GC development in mouse heterotopic xenograft model.** (**A**) CD47 expression in MKN45 cell lines after transfection with sh-CD47 plasmid. (**B**) Bioluminescence images of MKN45 tumors 60 days after engraftment. (**C**) Relative luciferase intensity of MKN45 cell lines after transfection with sh-CD47 plasmid (n = 5 for each group**;** **P < 0.01, paired *t-*test).

To explore the role of CD47 in GC development in mouse livers, we knocked down *CD47* in MNK45-luc2-labeled cancer cells and injected them into *BALB/c* nude mouse livers (Figure 2A). After 3 weeks, the tumor burden was estimated via bioluminescence. The results showed that, compared with that in mice of the sh-NC group (control), tumor bioluminescence was significantly low in mice of the sh-CD47 group (bioluminescence difference = -3.82e8 photos/s in shCD47 1#, -2.02e8 in shCD47 2#, **P < 0.01) (Figure 2B, C). Collectively, these results indicate that *CD47* knockdown inhibits GC progression in the liver tissues *in vivo*.

### *CD47* knockdown promotes cytokine secretion by macrophages

**Figure 3:**
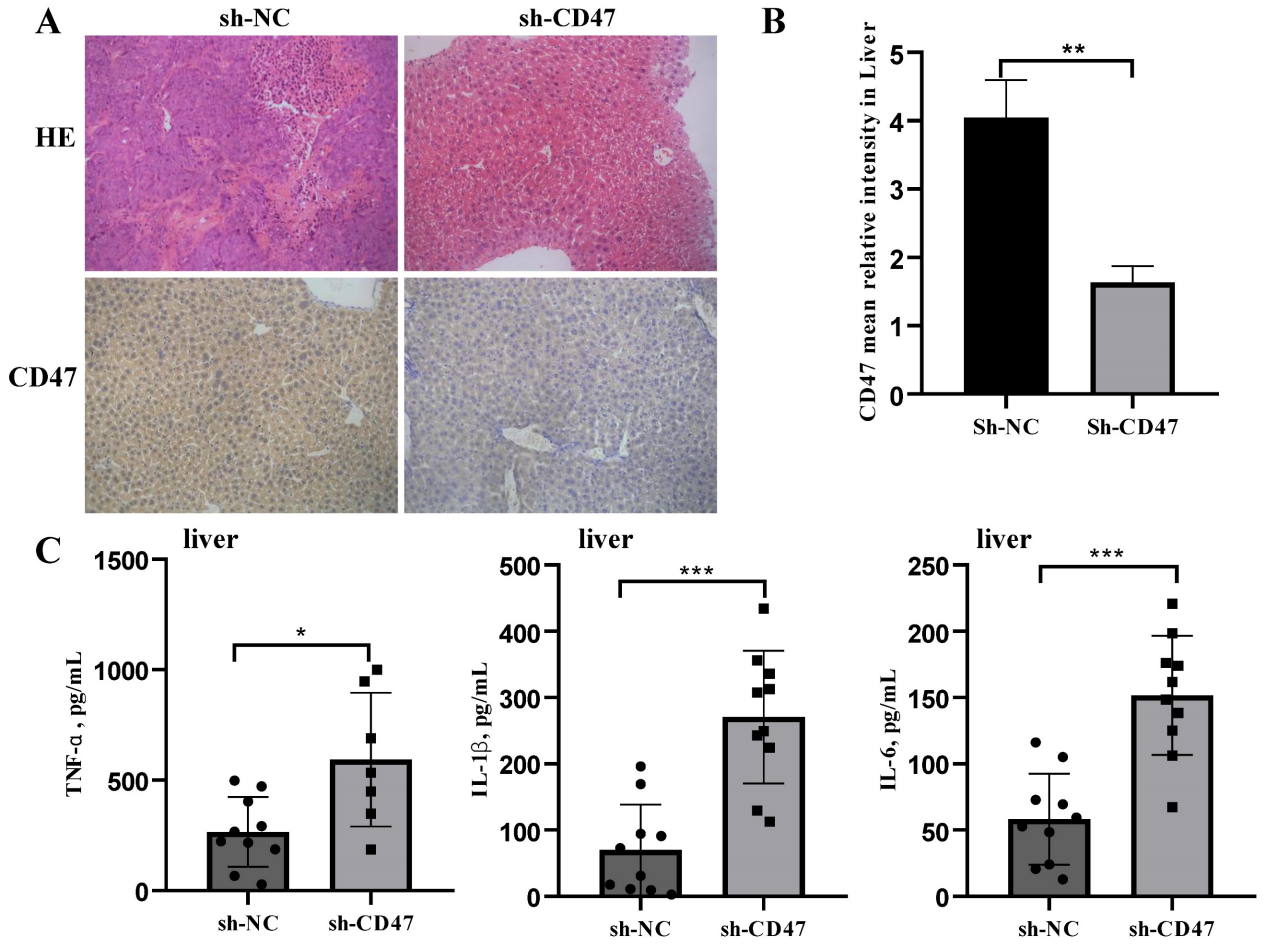
***CD47* knockdown increases the expression of cytokines.** (**A**) Hematoxylin and eosin and immunohistochemistry staining of metastatic liver of the cell-derived xenograft (CDX) model. (**B**) CD47 expression was higher in the sh-NC group than in the sh-CD47 group (n=10 for group, **P < 0.01, paired *t-*test). (**C**) TNF-α, IL-6, and IL-1β expression in the metastatic liver of the CDX model. (n = 10 for each group; *P < 0.05, **P < 0.01, ***P < 0.001, paired *t-*test).

We next investigated the effect of *CD47* knockdown on macrophage activation by analyzing cytokine secretion. Cell-derived xenograft (CDX) liver tissues were collected, homogenized, and subjected to centrifugation, followed by collection of supernatants and a multiplex analysis for evaluating different cytokines. CD47 expression in the CDX liver tissues was measured using immunohistochemistry. The results showed that CD47 expression in the sh-CD47 group was significantly higher than that in the sh-NC group (Figure 3A, B). We identified three mouse cytokines—TNF-α, IL-β, and IL-6—using ELISA. The levels of all cytokines were significantly increased in response to *CD47* knockdown (Figure 3C). These findings suggest that *CD47* knockdown promotes the secretion of cytokines and may increase macrophage recruitment, contributing to the efficacy of CD47-ablation therapies.

### *CD47* knockdown increases phagocytosis by KCs and FAK /PAK1/myosin phosphorylation in the CDX model

**Figure 4:**
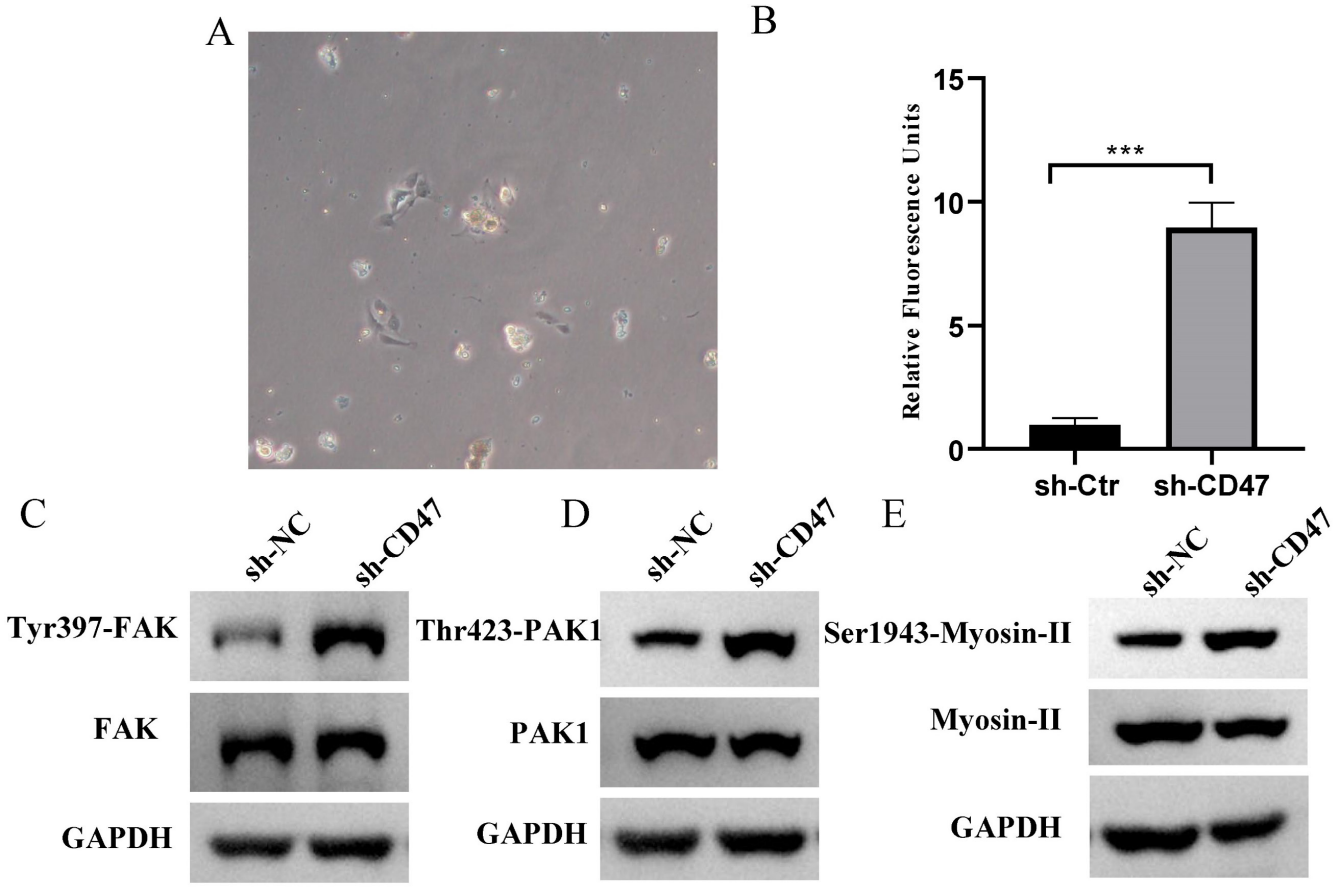
***CD47* knockdown promotes phagocytosis by Kupffer cells (KCs) and increases FAK phosphorylation in the CDX model.** (**A**) Morphology of isolated KCs in *BALB/c* nude mice. (**B**) Phagocytosis by KCs after co-culture with MNK45 cells. The extent of phagocytosis of MKN45 cells by KCs was quantified (n=3 for each group, ***P < 0.001, paired *t-*test). (**C-E**) Western blotting confirmed the phosphorylation of FAK, PAK1, and myosin.

Regarding the role of KCs in clearing GC cells, we hypothesized that KCs phagocytose GC cells and that the knockdown of *CD47* could increase the phagocytosis of GC cells by KCs. To test this hypothesis, we performed an engulfment assay *in vitro*. KCs isolated from the livers of *BALB/c* nude mice were co-cultured with sh-CD47 MKN45 or sh-NC MKN45 cells and subjected to flow cytometry for macrophage markers (Figure 4A). The results showed that sh-CD47 significantly enhanced MKN45 cell phagocytosis (Figure 4B) and promoted phagocytosis by sh-NC CDX KCs. These results suggest that *CD47* knockdown may accelerate the clearance of GC cells by enhancing phagocytosis by KCs. To further investigate the downstream pathways affected by *CD47* knockdown, we detected the expression of FAK, Tyr397-FAK, PAK1, Thr423-PAK1, myosin-II, and Ser1943-myosin in the CDX model. The supernatant of the MKN45 GC cell line was added to isolated KCs and protein levels were detected using western blotting. The Tyr397-FAK, Thr423-PAK1, Ser1943-myosin level was higher in the sh-CD47 group than in the sh-NC group; however, the levels of other molecules did not significantly differ between the groups (Figure 4C-E). These results indicate that inhibiting tumor growth through *CD47* knockdown involves upregulation of Tyr397-FAK, Thr423-PAK1, and Ser1943-myosin phosphorylation.

### Exosomes mediate phagocytosis by KCs

**Figure 5:**
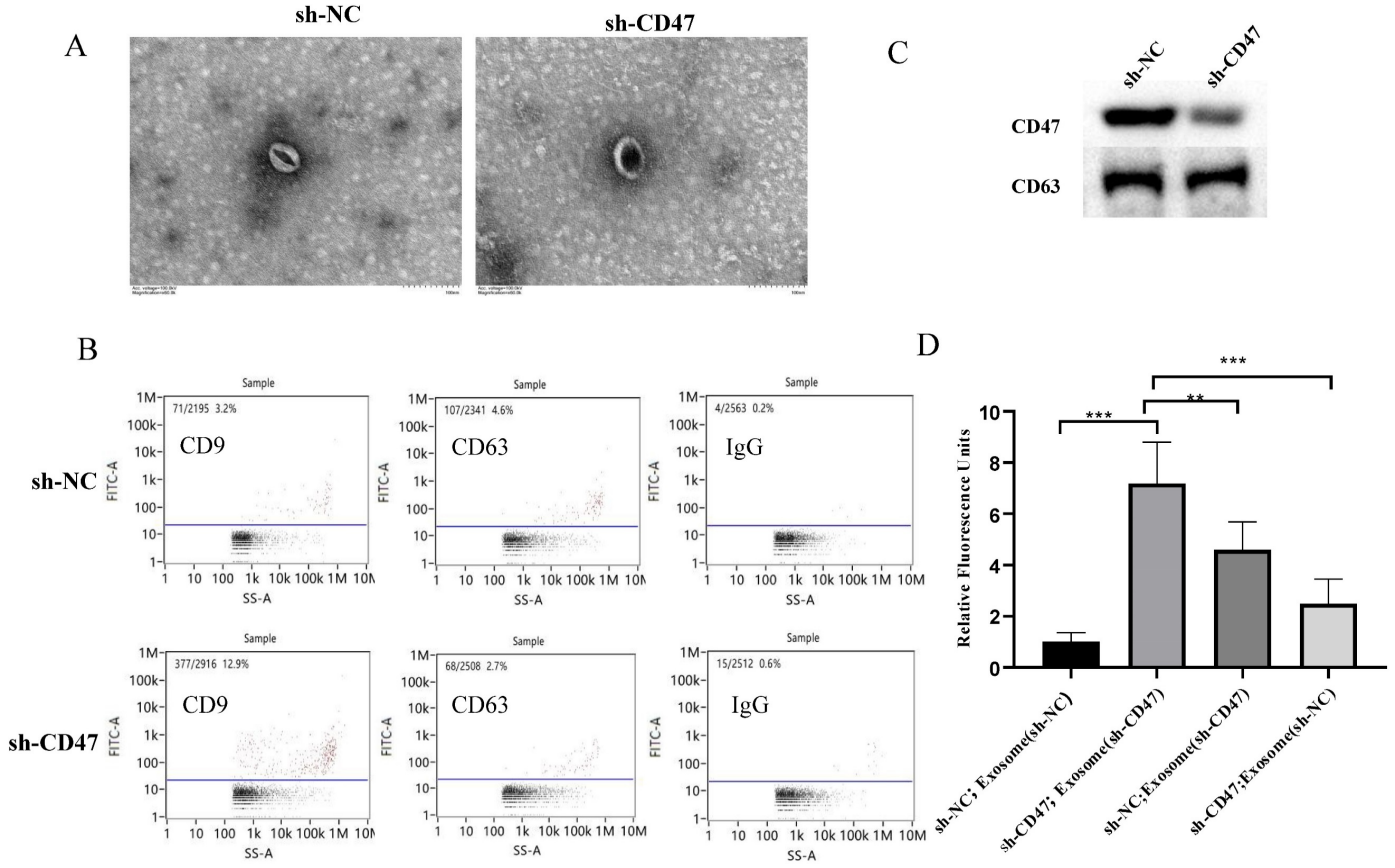
** Exosomes mediate phagocytosis by KCs.** (**A**) Transmission electron microscopy shows the morphology of exosomes (EXOs). Size distribution of EXOs. (**B**) Flow cytometry analysis of CD9 and CD63 expression on EXOs in the sh-NC or sh-CD47 group, respectively. (**C**) Expression of CD47 and CD63 on EXOs in the sh-NC or sh-CD47 group, respectively. (**D**) Extent of phagocytosis by macrophages in each group was quantified. The phagocytic index of the sh-CD47 group was the highest, followed by that of the sh-NCMKN45 cells and sh-CD47 EXO group and sh-CD47 MKN45 cells and sh-NC EXO group (n = 3 independent experiments, **P < 0.01, ***P < 0.001, n.s., no significance; one-way ANOVA).

To further explore how *CD47* knockdown enhances phagocytosis by KCs, we investigated the role of exosomes in this process. The sh-CD47 and sh-NC groups of MNK45-derived exosomes were examined using transmission electron microscopy, which revealed round particles with high homogeneity (Figure 5A). The flow cytometry analysis of the exosomes showed that the expression level of the exosome biomarkers CD63 and CD9 was 12.9% and 2.7% in the sh-CD47 group and 3.2% and 4.6% in the sh-NC group, respectively (Figure 5B). We then detected the expression of CD47 and CD63 in exosomes in the sh-CD47 and sh-NC groups, respectively. The results showed that CD47 expression was considerably lower in sh-CD47 cell exosomes than in sh-NC exosomes. However, no difference was observed in the expression of CD63 (Figure 5C) between the groups. To determine the effect of exosomes on phagocytosis, we treated KCs with carboxyfluorescein succinimidyl ester and co-cultured them with MKN45 cells and exosomes. Subsequently, the cells were evaluated using confocal microscopy. The deletion of CD47 in exosomes with sh-NC MNK45 cells increased the phagocytic index, which was higher than that in the sh-NC group. In addition, CD47 deletion in MKN45 cells with sh-NC exosome increased the phagocytic index compared with that in the sh-NC group (Figure 5D). Furthermore, the phagocytic index was the highest in sh-CD47 MKN45 cells with sh-CD47 exosomes, followed by the sh-NC MKN45 cells with sh-CD47 exosomes, and sh-CD47 MKN45 cells with sh-NC exosomes. The phagocytic index was the lowest in the sh-NC group (**P < 0.01, ***P < 0.001). Collectively, these results indicate that the exosomes mediated the phagocytosis of GCs by KCs.

### Combining 5-Fu with anti-CD47 antibody results in synergistic tumor suppression *in vivo*

**Figure 6:**
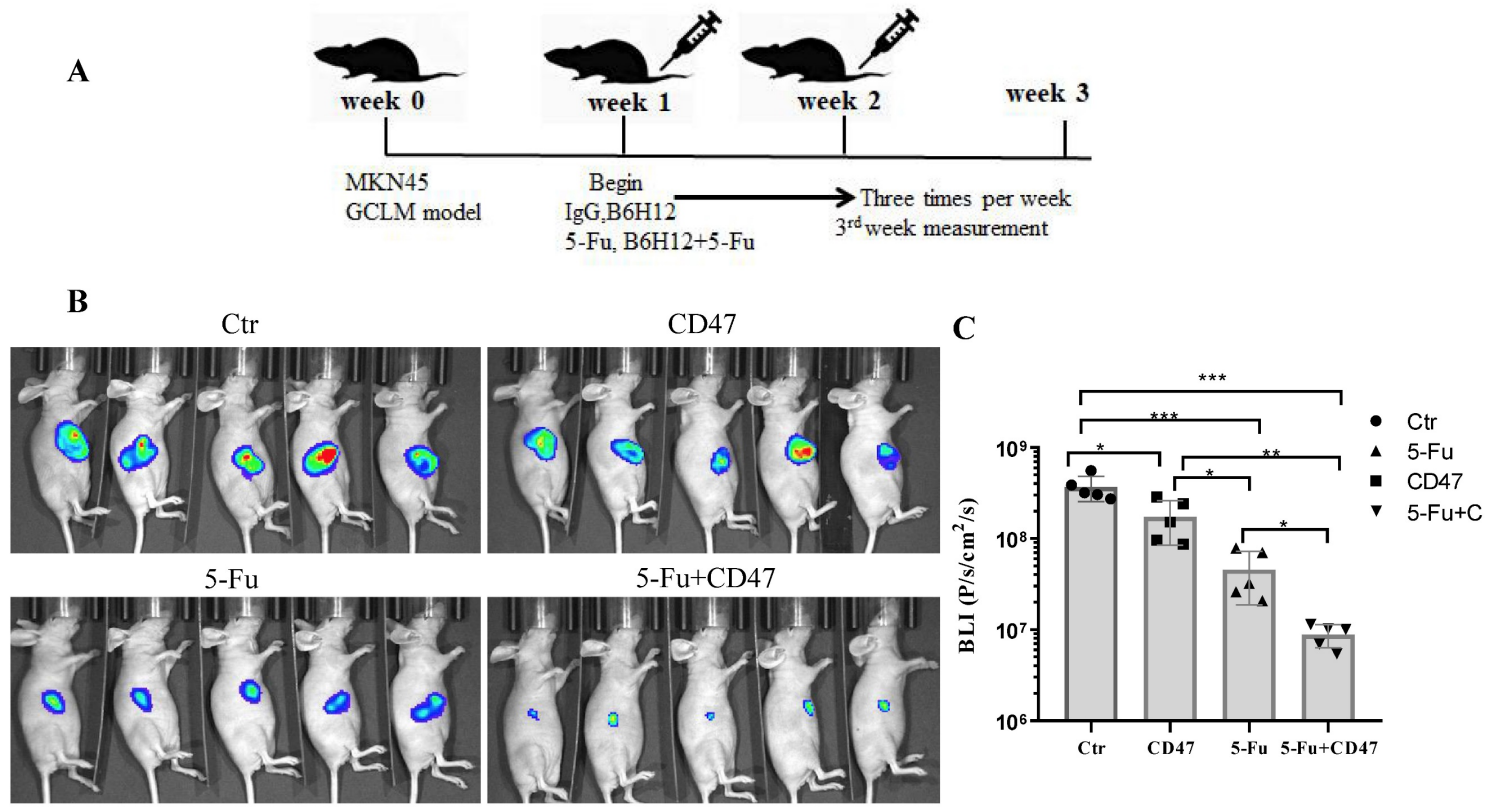
** CD47 antibodies inhibit tumor growth in the CDX model.** (**A**) MKN45 cells were implanted into the spleen of *BALB/c* nude mice and allowed to grow for 1 week, followed by injection of drugs (IgG and anti-CD47 antibody B6H12, tail injection; 5-Fu, intraperitoneal injection, or combination of B6H12 and 5-Fu) three times per week. (**B**) Bioluminescence images of mice at week 4. (**C**) Treatment with B6H12 antibodies + 5-Fu reduced tumor bioluminescence in the mice compared to treatment with IgG antibodies or CD47 antibodies or 5-Fu alone (n = 5 for each group; **P < 0.01, ***P < 0.001, one way ANOVA).

To validate the potential efficacy of blocking CD47 *in vivo*, we implanted MKN45-luc2-labeled tumor cells into *BALB/c* nude mouse spleens (Figure 6A). Approximately 1 week after engraftment, the mice were randomly assigned into four groups: with IgG control, anti-CD47 antibody B6H12, 5-Fu, or B6H12 and 5-Fu combination administered three times per week. After 2 weeks of treatment, the tumor burden was evaluated using bioluminescence. Tumor bioluminescence was lower in mice in which CD47 was blocked with B6H12 or 5-Fu (*P < 0.05, **P < 0.01) than in IgG-treated (control) mice. The tumor growth-inhibitory effect of the anti-CD47 antibody was similar to that of 5-Fu. Additionally, tumor growth was significantly decreased in 5-Fu and B6H12 combination-treated mice compared to that in control and single-treatment mice (***P < 0.001; Figure 6B, C). These results suggest that anti-CD47 antibody combined with 5-Fu shows a synergistic effect in suppressing tumors *in vivo*.

## Discussion

The innate immune system plays a critical role in clearing malignant cells and tumor-mediated immune escape. In various cancers, CD47 acts as a self-signal to inhibit phagocytosis using interacting with the macrophage receptor SIRPα 11,18. CD47 is expressed in various solid tumors, and anti-CD47 blockade inhibits tumor growth* in vitro* and *in vivo.* Sudo et al. showed that CD47 is overexpressed in cancer tissues compared to that in the adjacent tissues of patients with GC and that patients with high CD47 expression in the peripheral blood have a higher risk of lymphatic metastasis 19. A higher level of CD47 is associated with a poor prognosis, including recurrence and metastasis 12,20. Here, we showed that CD47 mRNA and protein expression was significantly higher in GCLM than in GC and the corresponding adjacent tissues. Furthermore, we demonstrated that patients with high CD47 expression showed a poor prognosis, indicating that cancer cells in GCLM escape phagocytosis by increasing CD47 expression.

KCs are the major macrophages in the liver and play important roles in the phagocytosis, detection, and elimination of apoptotic cells, commensal bacteria, tumor cells, and other antigens 6. In the early phases of metastatic liver colonization, KCs can induce inflammation and exert an important anti-metastatic role 6. We aimed to determine whether downregulating CD47 expression could inhibit the growth of GC cells in the liver and to reveal its effect on the phagocytosis of GC cells by KCs. Therefore, we conducted *in vivo* experiments to investigate how CD47 affects the growth of GC cell lines in mouse liver. Using a xenograft model, we transfected MKN45 cells with plasmid knockdown of *CD47*, and directly injected them into the liver to generate a CDX model. We found that sh-CD47 significantly inhibited GC growth in the mouse livers compared with that in control sh-NC mouse livers. This may be because sh-CD47 enhances MKN45 cell phagocytosis by KCs. Previously, Alex et al. demonstrated that CD47 blockade increases *in vitro* pancreatic ductal adenocarcinoma cell engulfment by macrophages 8. To examine the effect of *CD47* knockdown on phagocytosis by KCs, we co-cultured KCs isolated from mouse livers with sh-CD47 MKN45 cells. Sh-CD47 increased macrophage activity *in vitro*. Moreover, *CD47* knockdown decreased GC growth in the liver and enhanced phagocytosis by KCs.

GC-derived exosomes may have a pro-metastatic role in establishing a hospitable liver microenvironment 21,22. Exosome-derived epidermal growth factor receptor promotes the secretion of hepatocyte growth factors, which have important effects on the rate of GC metastasis 23. KCs play a key role in the development of a premetastatic niche in the liver. Therefore, we hypothesized that exosome-derived CD47 inhibits phagocytosis by KCs. We found that sh-NC MKN45 cells in combination with sh-CD47 exosomes increased phagocytosis by KCs *in vitro*, compared to the effects of sh-NC MKN45 cells with sh-NC exosomes. Interestingly, sh-NC MKN45 cells with sh-CD47 exosomes also increased phagocytosis by KCs compared with that in the NC group, indicating that GC-derived exosomes play an important role in mediating the function of KC in GCLM.

In addition to the phagocytic activity of KCs, inflammatory cytokine release contributes to their tumoricidal activity 24. In cholangiocarcinoma, anti-CD47 treatment potentiates M1 and M2 phagocytosis 25. To investigate whether the knockdown of *CD47* affects inflammatory cytokines, we co-cultured liver cells extracted from the CDX model with tumor cells to detect cytokine expression in the culture supernatant. We found that TNF-α, IL-β, and IL-6 expression was increased, in turn increasing neutrophil recruitment, followed by attracting and activating other immune cells. CD47 and SIRPα interact to activate tyrosine phosphatases and inhibit myosin accumulation, preventing phagocytosis. Here, *CD47* knockdown increased Ser1943-myosin-II phosphorylation, leading to the promotion of cytoskeleton rearrangement as a crucial step for macrophages to engulf target cells 26.

Xenograft mouse models have been used in studies to evaluate the anticancer effects of CD47 blockade, and the results have revealed a significant inhibitory effect on various cancers 27-29. Targeting CD47 alone has been efficacious in several preclinical tumor models 30; however, combination strategies offer increased therapeutic potential. Non-small cell lung cancer is sensitive to anti-angiogenic therapy when CD47 is blocked, and macrophages can better infiltrate the tumor and kill it 31. An anti-CD47 antibody in combination with doxorubicin leads to synergistic elimination of hepatocellular carcinoma in mice 32. 5-Fu alone or in combination with other conventional therapies has become the standard chemotherapy regimen 33. Our *in vitro* experiments of the combined effect of anti-CD47 antibody and 5-Fu in MKN45 cell-bearing mice was positive; however, anti-CD47 antibodies alone marginally reduced the tumor size, whereas 5-Fu combined with anti-CD47 antibodies significantly reduced the tumor size, suggesting a synergistic effect of the combination treatment. Importantly, the combination therapy improved the therapeutic efficacy of 5-Fu.

## Conclusions

We found that *CD47* knockdown inhibited GC growth in the liver and enhanced macrophage-mediated phagocytosis. We determined that tumor-derived exosomes were involved in phagocytosis by KCs. More importantly, anti-CD47 antibodies were found to inhibit *in vivo* tumor growth and exerted a synergistic effect with 5-Fu treatment. Overall, combination therapy with anti-CD47 antibodies and 5-Fu showed potential for treating GCLM.

## Abbreviations

5-Fu: 5-fluorouracil
ADGC: adjacent normal gastric tissue
ADLM: adjacent normal liver tissue
ANOVA: analysis of variance
CDX: cell-derived xenograft
ELISA: enzyme-linked immunosorbent assay
EXO: exosome
GC: gastric cancer
GCLM: gastric cancer liver metastasis
GCLM: gastric cancer liver metastasis
IL: interleukin
KC: Kupffer cell
OS: overall survival
PBS: phosphate-buffered saline
Sh: short hairpin
TNF: tumor necrosis factor
